# Orthopedic Device-Related Infections Due to Emerging Pathogens Diagnosed by a Combination of Microbiological Approaches: Case Series and Literature Review

**DOI:** 10.3390/diagnostics12123224

**Published:** 2022-12-19

**Authors:** Angela Quirino, Nadia Marascio, Giuseppe Guido Maria Scarlata, Claudia Cicino, Grazia Pavia, Marta Pantanella, Giovanni Carlisi, Michele Mercurio, Filippo Familiari, Salvatore Rotundo, Vincenzo Olivadese, Valentina La Gamba, Francesca Serapide, Giorgio Gasparini, Giovanni Matera

**Affiliations:** 1 Unit of Clinical Microbiology, Department of Health Sciences, “Magna Graecia” University, 88100 Catanzaro, Italy; 2 Unit of Orthopedic and Trauma Surgery, Department of Medical and Surgical Sciences, “Magna Græcia” University, 88100 Catanzaro, Italy; 3 Unit of Infectious and Tropical Diseases, Department of Medical and Surgical Sciences, “Magna Graecia” University, 88100 Catanzaro, Italy

**Keywords:** emerging pathogens, MALDI-TOF, 16SrRNA sequencing, *Abiotrophia defectiva*, *Finegoldia magna*

## Abstract

Orthopedic and trauma device-related infections (ODRI) due to high virulence microorganisms are a devastating complication after orthopedic surgery. Coagulase-negative Staphylococci (CoNS) are mainly involved but commensal bacteria, located in human mucous membranes, are emerging pathogens in ODRI. Currently, bacterial culture is the gold standard for ODRI but the diagnostic process remains time consuming and laborious. We evaluated a combination of microbiological approaches in the diagnosis of emerging pathogens involved in ODRI. We analyzed two synovial fluids, five tissue samples and five surgical wound swabs from two different patients with ODRI, attending the Department of Orthopedic and Trauma Surgery of *Mater Domini* Teaching Hospital, Catanzaro, Italy. Identification was carried out with a combination of microbiological approaches (culture, mass spectrometry and 16s rRNA gene sequencing). We demonstrated the importance of a combination of microbiological approaches for the diagnosis of emerging pathogens in ODRI, because the low number of cases in the literature makes it very difficult to formulate guidelines for the management of patients.

## 1. Introduction

Orthopedic and trauma device-related infections (ODRI) are one of the most challenging and devastating complications after orthopedic surgery. These infections are a burden on clinical impact and public costs and require special prevention strategies [1]. The involvement of high virulence microorganisms is responsible for the high mortality rate reported in patients with ODRI [2]. Two-thirds of ODRI are due to inoculation of microorganisms during surgery, such as *Staphylococcus aureus*, *Streptococcus* spp., *Enterococcus* spp. in early infections, while Coagulase-negative Staphylococci (CoNS) are mainly involved in delayed infections [3]. The clinical manifestations of ODRI are related to many factors, including pathogen virulence, host immune status, the soft tissue structure surrounding the joint and the joint involved. The main symptoms are pain, swelling or joint effusion, erythema or warmth around the joint, fever and drainage or presence of a sinus tract communicating with the arthroplasty [4]. In our experience, over a period of 10 years (between 1 January 2011 and 31 December 2021), the prevalence of microorganisms responsible for ODRI was as follows CoNS (35%), Staphylococcus aureus (26%), Enterobacteriaceae (24%), with the final 15% including *Abiotrophia defectiva*, *Finegoldia magna*, *Veillonella* spp., *Prevotella oralis*, *Gemella haemolysans* and *Granulicatella elegans.* The microorganisms included in the smallest group, are commensal bacteria located in human mucous membranes, such as the oral cavity, upper respiratory or gastrointestinal tract and they are sometimes involved in ODRI as emerging pathogens [5]. Currently, the diagnostic workflow remains long and difficult, leading to a considerable delay in patient treatment [6]. A serious problem relates to ODRIs diagnosed with a negative culture. This may be due to the inability to isolate the pathogen following previous antimicrobial therapy, inadequate use of available microbiological methods or the inability to detect a pathogen responsible for ODRI using currently available diagnostic methods [7]. The use of rapid and accurate tests for the identification of microorganisms is a priority in microbiological diagnostics. Although culture remains the gold standard for the diagnosis of bacterial infections, it can take several days to isolate pathogens and to assess their antimicrobial susceptibility. Genomic sequencing of small-subunit ribosomal RNA (16S rRNA) is often employed to identify and classify bacteria isolates, through assigning the sequence to a taxonomic bin based on known references either by classification or identification of the closest type of strain [8]. During the last few years, the application of matrix-assisted laser desorption ionization time of flight (MALDI-TOF) for identification purposes has proved a promising alternative for the rapid identification of bacterial isolates from clinical samples (which is more cost and time effective compared to 16srRNA sequencing) [9]. Therefore, rapid, and accurate diagnostic tests, combined with conventional methods, are useful for early and targeted therapy, improving clinical outcome [10]. For this reason, a combination of microbiological approaches are required for more accurate microbial identification and were applied in the following case series.

## 2. Materials and Methods

### 2.1. MALDI-TOF Vitek MS

Vitek MS (bioMérieux, Craponne, France) is an automated system based on mass spectrometry technology, which can detect genus and species of microorganisms in a very short time. A single bacterial colony was placed on a slide using a loop followed by the addition of 1 μL of the matrix (Vitek MS-CHCA) and air drying. The slide was inserted into the Vitek MS system for mass spectrum generation. The obtained spectra were compared with those in the Vitek MS database. The peak features were compared with those characteristics for genus and species of the database microorganisms for definitive identification. The microorganisms were reported with a percentage-scaled confidence value, as well as a confidence level. Escherichia coli ATCC 8739 was included as a positive control.

### 2.2. Vitek^®^2 System 

Vitek^®^2 system (bioMérieux, France) is a fully automated system that performs bacterial identification and antibiotic susceptibility testing using test cards, which contain dehydrated biochemical substrates. In these cases, Gram-positive cards were used for phenotypical identification.

### 2.3. The 16S rRNA Gene Sequencing 

Bacterial DNA extraction from pure culture was performed using QIAamp^®^ DNA Mini Kit (Qiagen, Hilden, Germany) followed by polymerase chain reaction (PCR) amplification. Identification was confirmed by 16SrRNA Sanger sequencing (ABI PRISM 3500 Genetic Analyzer, Applied Biosystems, Waltham, MA, USA). The sequences were edited by SeqScape Software Version 2.5 (Applied Biosystems, USA), then compared to the reference sequence available in the National Center for Biotechnology Information (ncbi.nlm.nih.gov, accessed on 26 September 2022) using the Basic Local Alignment Search Tool nucleotide (BLASTn) algorithm [11]. 

### 2.4. Antimicrobial Susceptibility Testing 

The minimal inhibitory concentrations (MICs) of amoxicillin, amoxicillin–clavulanic acid, ampicillin, ampicillin/sulbactam, cefoxitin, cefoxitin screen, ceftaroline, clindamycin, chloramphenicol, daptomycin, doxycycline, erythromycin, gentamicin, levofloxacin, linezolid, metronidazole, moxifloxacin, mupirocin, nitrofurantoin, penicillin, piperacillin, piperacillin-tazobactam, rifampicin, streptomycin, teicoplanin, tigecycline, trimethoprim/sulfamethoxazole and vancomycin were determined by Sensititre™ Gram positive MIC Plate and Anaerobe MIC Plate (Thermo Fisher Scientific, Waltham, MA, USA). Imipenem susceptibility was carried out by E-Test using Mueller–Hinton agar (37 °C, 24 h), according to Li J. et al., 2022 [12]. Susceptibility to antibiotics was interpreted based on the European Committee on Antimicrobial Susceptibility Testing (EUCAST) breakpoints.

## 3. Case Series

**Figure 1:**
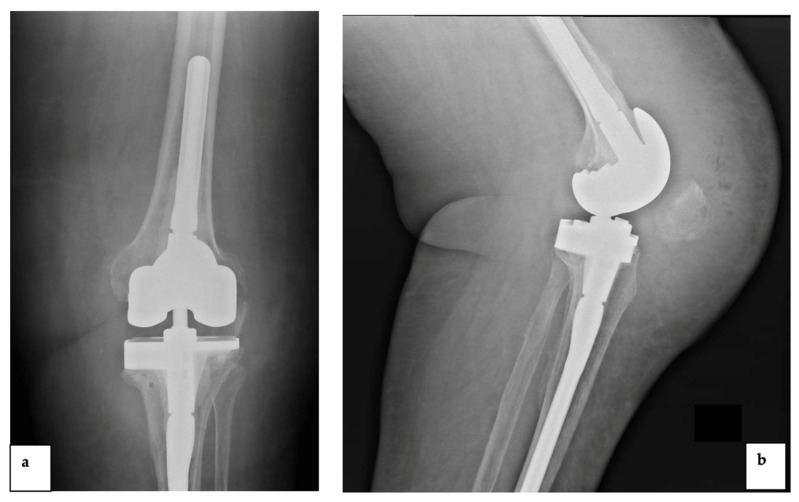
The plain radiograph showed periprosthetic infection of the condylar constrained knee implant due to *Staphylococcus warneri* ((**a**): anteroposterior view and (**b**): lateral view).

Patient 1 was a 69-year-old woman admitted to the Department of Orthopedic and Trauma Surgery of *Mater Domini* Teaching Hospital, Catanzaro, Italy, with the diagnosis of aseptic loosening of the left total knee arthroplasty, implanted 7 years previously. A one-stage revision arthroplasty was performed with a condylar constrained knee (CCK) implant. After four months, the patient experienced an accidental trauma with swelling of the left knee (Figure 1). 

**Figure 2:**
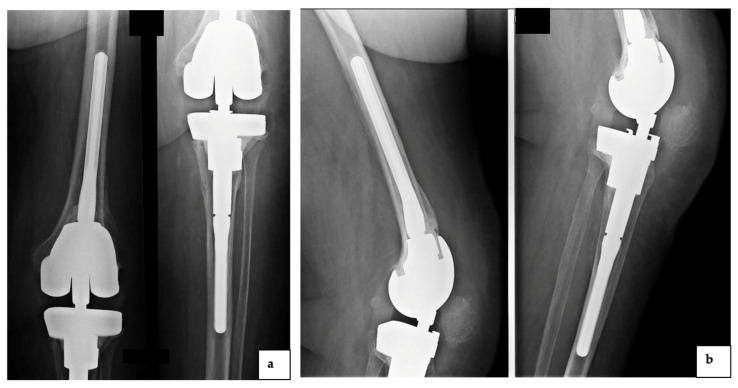
The plain radiograph showed periprosthetic infection of the rotating hinge knee implant due to *Abiotrophia defectiva* ((**a**): anteroposterior view and (**b**): lateral view).

Thirty days later, the patient referred to our institution due to persistence of pain and swelling and a synovial fluid analysis was performed for suspected ODRI. An early empiric intravenous antibiotic therapy was started with 1 g vancomycin two times daily, 600 mg rifampicin daily and 500 mg levofloxacin daily. Synovial fluid was positive for *Staphylococcus warneri* sensitive to the administered antibiotics. Subsequently, the implantation of an antibiotic spacer was performed, followed three months later by the re-implantation of a rotating hinge knee (RHK) prosthesis. Given the favorable clinical and laboratory evolution, the patient was discharged, and the same antibiotics were continued for six weeks until the inflammatory markers normalized. Four years after the second reimplantation, the patient reported pain and swelling during a routinely scheduled follow-up at our institution (Figure 2). 

**Figure 3:**
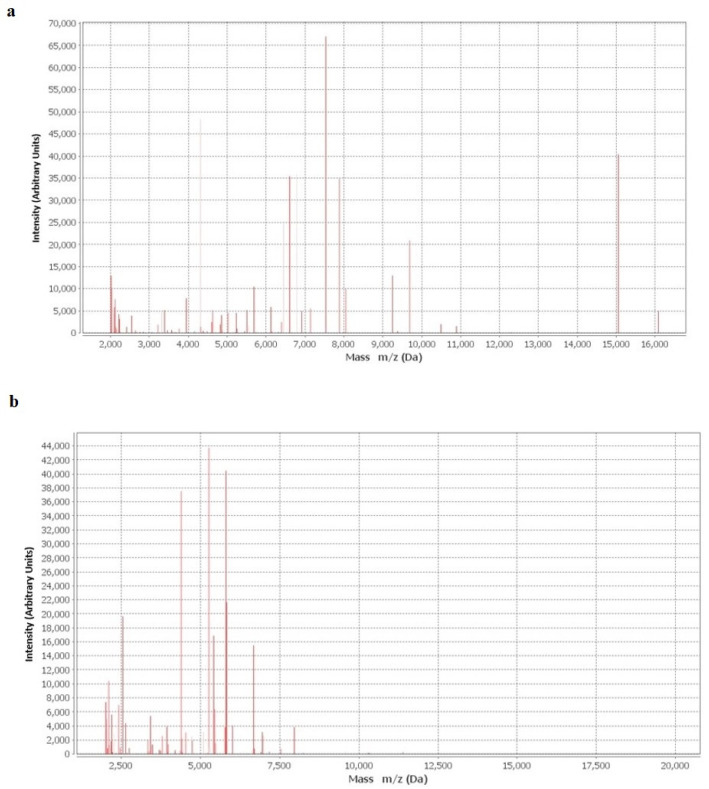
Mass spectra of Abiotrophia defectiva (**a**) and Finegoldia magna (**b**).

Synovial fluid culture was performed at baseline and 20 days later, by inoculation into aerobic and anaerobic blood culture bottles. After 40 h, the cultures were positive for Gram-positive, pleomorphic and coccus–bacilli arranged in couples or short chains seen by microscopy. *Abiotrophia defectiva* from pure anaerobic culture was identified by both Vitek MS (bioMérieux, France) (Figure 3a) and Vitek^®^2 (bioMérieux, France). The 16SrRNA gene exhibited high similarity to *Abiotrophia defectiva* (accession number GIFU 12707, ATCC 49176). Imipenem susceptibility showed a 0.008 mg/L minimum inhibitory concentration (MIC) value.

Antimicrobial susceptibility by broth microdilution showed resistance to levofloxacin (Table 1). Subsequently, intravenous antibiotic therapy with 3 g ampicillin four times daily and 2 g of ceftriaxone daily was started. The patient refused further surgical procedures. After three weeks, given the favorable clinical and laboratory evolution, the patient was discharged and oral therapy with 2 g amoxicillin two times daily and 100 g doxycycline two times daily was continued for 6 weeks until the inflammatory markers normalized. Close clinical and laboratory follow-up was performed monthly. Five months after discharge from the hospital, the inflammatory markers were still normal, and no complications or recurrence of the disease were reported.

**Figure 4:**
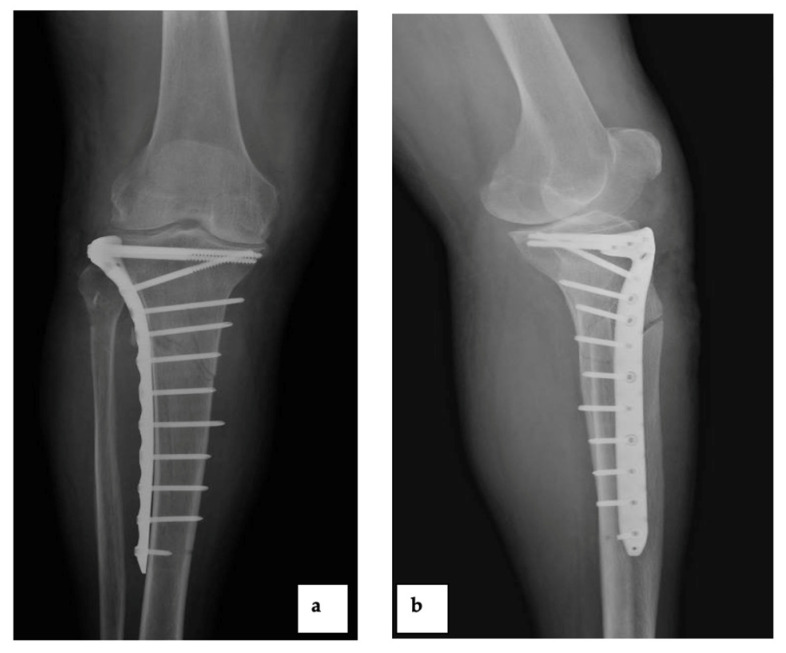
The plain radiograph showed the infected tibial plate ((**a**): anteroposterior view and (**b**): lateral view).

Patient 2 was a 67-year-old man, with a previous infection due to *Staphylococcus aureus* after osteosynthesis surgery following a tibia and fibula fracture and readmitted 3 months later to the Department of Orthopedic and Trauma Surgery of our institution, because of pain in the previously implanted tibial plate (Figure 4). 

**Figure 5:**
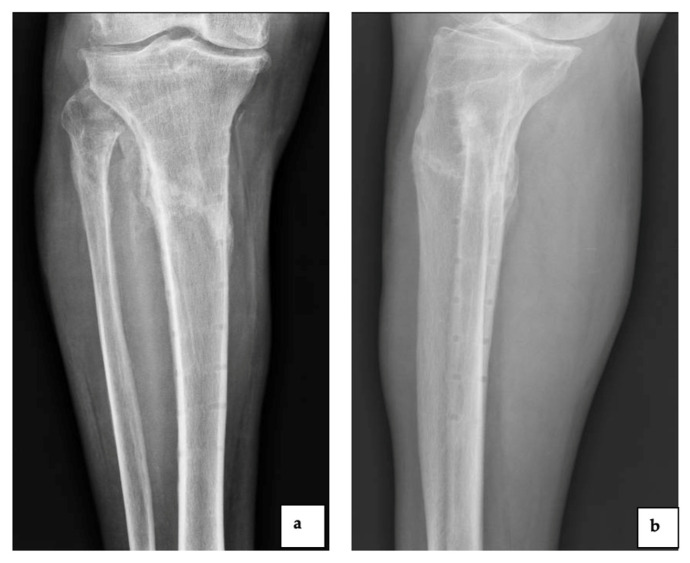
The plain radiograph after removal of the tibial plate at 6 months of follow-up ((**a**): anteroposterior view and (**b**): lateral view).

Five surgical wound swabs and five tissue samples were cultured on Columbia Blood Agar (CBA), MacConkey Agar and Sabouraud Dextrose Agar (SDA) at 37 °C under aerobic conditions for five days and in CBA at 37 °C under anaerobic conditions for the same period. *Finegoldia magna* from pure anaerobic culture was identified by both mass spectrometry (Figure 3b) and Vitek^®^2 (bioMérieux, France). Identification was confirmed by 16SrRNA Sanger sequencing (ABI PRISM 3500 Genetic Analyzer, Applied Biosystems) and displayed high similarity with *Finegoldia magna* (accession number CP054000.1). Resistance testing was carried out by Vitek^®^2 (bioMérieux, France) and Sensititre™ (Thermo Fisher Scientific, USA), showing resistance to linezolid and rifampicin (Table 1). Antibiotic therapy with cefazolin 2 g two times daily was administered, followed by surgery to remove the tibial plate. Subsequently, the patient was discharged and submitted to follow-up, without reporting complications or recurrence of the disease (Figure 5). 

## 4. Discussion

Conventional microbiological methods are used to identify bacteria isolates from clinical samples based on microscopic observation, analysis of cultural and metabolic characteristics using biochemical tests, miniaturized galleries and automated methods. Although culture remains the gold standard for the diagnosis of bacterial infections, conventional procedures of identification have long turn-around times, while diagnosis needs to be faster to improve patient care. The implementation of rapid and accurate diagnostic tests, such as MALDI-TOF, combined with conventional methods, was useful for more accurate microbial identification and early targeted therapy, improving clinical outcome. Currently, two main commercial systems are available on the market: the Vitek MS (bioMérieux, France) and the Microflex LT Biotyper (Bruker Daltonics, Billerica, MA, USA). In both systems, anaerobic bacteria were identified with a high level of confidence and the few errors were due to the lack of a commercial database containing representative isolates for the most-frequent clinically relevant anaerobic species. These phenotypic methods were designed to speed up culture-based diagnosis and to combine the highest value for accuracy, range of identification, cost-effectiveness and timely diagnosis for critical patients and better antimicrobial stewardship [13]. Specifically, the use of MALDI-TOF allows mono- or polymicrobial infections to be identified in a short time, in comparison to conventional identification methods, such as the Vitek^®^2 System [14]. The turnaround time for microbiological identification with MALDI-TOF is about 25 min, whereas subculture followed by identification with conventional methods takes about 48–72 h [15]. Furthermore, the Vitek^®^2 System requires continuous updating of its database, in order to detect the less frequently isolated microorganisms from clinical samples [16]. The 16SrRNA gene sequencing has been accepted as the reference method for species identification, despite higher costs and the need for experienced personnel [17]. 

Our study shows a good accuracy between Vitek MS and 16SrRNA gene sequencing to detect emerging pathogens, such as *Abiotrophia defectiva* and *Finegoldia magna*. Early and accurate identification allowed a diagnosis of ODRI, leading to the use of targeted antibiotic therapy, in a short time. In both cases therapeutic success was made possible by the rapid and accurate identification of the pathogens. Ratcliffe P. and co-workers compared the Vitek MS and Vitek^®^2 systems for the identification of *Abiotrophia defectiva* and *Granulicatella elegans* in blood samples, showing a higher accuracy of Vitek MS (14/14 identified isolates) versus Vitek^®^2 (9/14 identified isolates). Furthermore, definitive identification was performed by 16SrRNA gene sequencing, used as the diagnostic gold standard in the study [18]. The best combination of sensitivity and specificity was obtained when Marin M. and colleagues analyzed synovial fluid samples by culture and 16SrRNA, to identify pathogens involved in ODRI, such as *Finegoldia magna* and *Gemella morbillorum* [19]. This case should draw attention to unusual commensal opportunistic bacteria in ODRI. Indeed, there are very few published clinical reports in the orthopedic field due to *Abiotrophia defectiva* and *Finegoldia magna*, as shown in Table 2. 

Interestingly, we observed just two cases of re-infection at the same site by a different pathogen [3,26] since it is usually reported that high virulence microorganisms can promote reinfection with the same pathogen [27]. Here, we reported a re-infection at the same site by *Abiotrophia defectiva* and *Finegoldia magna* after successfully treating *Staphylococcus warneri* and *Staphylococcus aureus* infections, respectively. 

## 5. Conclusions

In conclusion, we demonstrated the importance of using a combination of microbiological approaches for the diagnosis of emerging pathogens in ODRI, since the low number of cases in the literature makes it very difficult to formulate guidelines for the management of patients. A multidisciplinary approach is necessary for a timely diagnosis and to develop the most effective eradication strategy, whilst providing fewer complications [28].

## Figures and Tables

**Table 1:** Antimicrobial susceptibility test of *Abiotrophia defectiva* and *Finegoldia magna* isolates.

	*Abiotrophia defectiva*		*Finegoldia magna*	
Antimicrobial Drug	MIC (mg/L)	Interpretation	MIC (mg/L)	Interpretation
Amoxicillin	-	-	≤0.25	S
Amoxicillin-clavulanic acid	-	-	≤0.25	S
Ampicillin	2	S	-	-
Ampicillin/sulbactam	≤2	S	-	-
Cefoxitin	≤4	nd	-	-
Cefoxitin screen	≤6	nd	-	-
Ceftaroline	≤0.12	nd	-	-
Clindamycin	≤0.12	nd	2	S
Chloramphenicol	-	-	≤2	S
Daptomycin	1	nd	-	-
Doxycicline	1	nd	-	-
Erythromycin	≤1	nd	-	-
Gentamicin	2	nd	-	-
Imipenem	-	-	≤0.12	S
Levofloxacin	>4	R	-	-
Linezolid	≤0.5	S	≤1	R
Metronidazole	-	-	S	S
Moxifloxacin	-	-	≤0.12	nd
Mupirocin	>256	nd	-	-
Nitrofurantoin	>64	nd	-	-
Penicillin	-	-	0.12	S
Piperacillin	-	-	≤2	S
Piperacillin-Tazobactam	-	-	≤2	S
Rifampicin	≤0.06	nd	≤1	R
Streptomycin	>512	nd	-	-
Teicoplanin	0.5	nd	-	-
Tigecycline	0.12	S	≤1	nd
Trimethoprim/Sulfamethoxazole	≤0.25	nd	-	-
Vancomicyn	≤0.5	nd	≤1	S

Legend: S: sensitive; R: resistant; nd = breakpoint not determined.

**Table 2:** Literature review on *Abiotrophia defectiva* and *Finegoldia magna* in the orthopedic field.

Patient Age, Sex	Clinical Features	Microbiological Highlights	Treatment	Reference
65 years, F	Progressive pain and swelling in right knee.	Cultured synovial fluid on chocolate agar incubating at 37 °C for 72 h in an atmosphere containing 5–10% CO_2_, *Abiotrophia defectiva* was identified by sequencing; antibiogram was performed by disk diffusion.	Two-stage revision arthroplasty; cefazolin i.v. for 10 days and ciprofloxacin orally for 26 days.	Ince et al., 2002 [20]
71 years, M	Chronic left knee pain, swelling and decreasing ambulation.	Cultured synovial fluid on chocolate agar incubating at 37 °C in an atmosphere containing 5–10% CO_2_, *Abiotrophia defectiva* was identified by sequencing; antibiogram was performed by E-Test.	Inserting of temporary cement spacer containing 2 g vancomycin and 40 g cement followed by re-implantation of a total knee; 100 mg/kg/day oral amoxicillin for nine months.	Cassir N. et al., 2011 [21]
74 years, M	Knee pain and inability to ambulate. Previous ODRI due to Methicillin-resistant Staphylococcus epidermidis (MRSE).	*Abiotrophia defectiva* was isolated from synovial fluid and tissue samples after 5 days of incubation under aerobic condition and identified by mass spectrometry.	Cefrtriaxone i.v. for 6 weeks and 3 months after, antibiotic-impregnated cement, containing six packages of tobramycin and two packages of vancomycin powder (ratio of 3:1) was implemented. Postoperatively, the patient was started on oral cephalexin (500 mg/3 daily) for 3 months.	Tooley TR et al., 2019 [3]
65 years, M	Progressive knee pain and swelling bilaterally, apyretic until third day of hospitalisation.	*Abiotrophia defectiva* was isolated by three blood cultures; unable to achieve sufficient growth for antibiotic sensitivity.	Simultaneous bilateral 2-stage revision with articulated cement spacers impregnated with vancomycin and gentamycin; 6 weeks of i.v. antibiotics after each stage.	Wan J et al., 2020 [22]
69 years, F	Swelling and knee pain.	Cultured synovial fluid on chocolate agar with supplemented pyridoxal 37 °C under a 5% CO_2_ atmosphere for 24 h; *Abiotrophia defectiva* was identified by mass spectrometry; antibiogram was performed by disk diffusion on Mueller–Hinton agar with pyridoxal-supplemented sheep blood (CO_2_ 5%, 37 °C, 24 h).	2-stage revision arthroplasty; 4 × 1000.000 IU/mL penicillin G and 3 × 80 mg/L gentamicin IV had been administered parenterally for 30 days.	Kocazeybek E et al., 2020 [23]
71 years, M	Swelling, knee pain and difficulty walking. Previous left knee revision due to an ODRI with an unknown etiology three years prior.	*Abiotrophia defectiva* from synovial fluid and blood culture was identified by sequencing. Sensitivities could not be performed as the bacteria was not viable for susceptibility testing.	Cefepime i.v. 2 g three times a day, switched to ceftriaxone i.v. 2 g for six weeks.	Young J.N. et al., 2022 [24]
65 years, M	Pain in the left hip after having undergone arthroplasty three years prior.	*Finegoldia magna* was isolated from intraoperative material and identified by mass spectrometry.	Piperacillin/tazobactam 4 times a day, 4.5 g intravenously, over 7 days.	Szymczak Z. et al., 2017 [25]
55 years, F	Recurrent exudates in left trochlear bursa which arose 5 years after left hip arthroplasty.	*Finegoldia magna* was isolated from surgical swabs and identified by mass spectrometry.	Piperacillin/tazobactam 4 times a day, 4.5 g intravenously, over 7 days	Szymczak Z. et al., 2017 [25]
52 years, F	Previous polymicrobial ODRI due to *Cutibacterium avidum* and *Citrobacter koseri* after surgical debridement of all infected tissues and explanation of the prosthesis.	*Finegoldia magna* was identified by mass spectrometry; whole-genome sequencing was performed to classified as “wild-type” or “non-wild- type”.	Joint prosthesis was explanted and intravenous antibiotic treatment with amoxicillin was initiated for two weeks followed by metronidazole per day for four weeks prior to implantation of a new hip prosthesis.	Walser F. et al., 2022 [26]

## Data Availability

The newly generated sequences can be retrieved under GenBank^®^ accession numbers ON123605 and ON974995.

## References

[1] Moriarty T.F., Kuehl R., Coenye T., Metsemakers W.-J., Morgenstern M., Schwarz E.M., Riool M., Zaat S.A.J., Khana N., Kates S.L. (2017). Orthopaedic device-related infection: Current and future interventions for improved prevention and treatment. EFORT Open Rev..

[2] Izakovicova P., Borens O., Trampuz A. (2019). Periprosthetic joint infection: Current concepts and outlook. EFORT Open Rev..

[3] Tooley T.R., Siljander M.P., Hubers M. (2019). Development of a periprosthetic joint infection by Abiotrophia defectiva years after total knee arthroplasty. Arthroplast. Today.

[4] Tande A.J., Patel R. (2014). Prosthetic joint infection. Clin. Microbiol. Rev..

[5] Deppe H., Ritschl L.M., Vacha E., Rechl H., Wantia N., Wagenpfeil S., Sculean A. (2019). Periodontopathogenic bacteria in prosthetic joint infection: A retrospective analysis of 1,673 patients. Quintessence Int..

[6] Kim S.J., Cho Y.J. (2021). Current Guideline for Diagnosis of Periprosthetic Joint Infection: A Review Article. Hip Pelvis.

[7] Cazanave C., Greenwood-Quaintance K.E., Hanssen A.D., Karau M.J., Schmidt S.M., Gomez Urena E.O., Madrekar J.N., Osmon D.R., Lough L.E., Pritt B.S. (2013). Rapid molecular microbiologic diagnosis of prosthetic joint infection. J. Clin. Microbiol..

[8] Kim O.-S., Cho Y.-J., Lee K., Yoon S.-H., Kim M., Na H., Park S.-C., Jeon Y.S., Lee J.-H., Yi H. (2012). Introducing EzTaxon-e: A prokaryotic 16S rRNA gene sequence database with phylotypes that represent uncultured species. Int. J. Syst. Evol. Microbiol..

[9] Seng P., Abat C., Rolain J.M., Colson P., Lagier J.-C., Gouriet F., Fournier P.E., Drancourt M., La Scola B., Raoult D. (2013). Identification of rare pathogenic bacteria in a clinical microbiology laboratory: Impact of matrix-assisted laser desorption ionization-time of flight mass spectrometry. J. Clin. Microbiol..

[10] Jamal W., Al Roomi E., Abdul Aziz L.R., Rotimi V.O. (2014). Evaluation of Curetis Unyvero, a multiplex PCR-based testing system, for rapid detection of bacteria and antibiotic resistance and impact of the assay on management of severe nosocomial pneumonia. J. Clin. Microbiol..

[11] Zhang Z., Schwartz S., Wagner L., Miller W. (2000). A greedy algorithm for aligning DNA sequences. J. Comput. Biol..

[12] Li J., Zhou L., Gong X., Wang Y., Yao D., Li H. (2022). Abiotrophia Defectiva as a Rare Cause of Mitral Valve Infective Endocarditis With Mesenteric Arterial Branch Pseudoaneurysm, Splenic Infarction, and Renal Infarction: A Case Report. Front. Med..

[13] Benagli C., Rossi V., Dolina M., Tonolla M., Petrini O. (2011). Matrix-assisted laser desorption ionization-time of flight mass spectrometry for the identification of clinically relevant bacteria. PLoS ONE.

[14] Kuo F.C., Chien C.C., Lee M.S., Wang J.W., Lin P.C., Lee C.H. (2020). Rapid diagnosis of periprosthetic joint infection from synovial fluid in blood culture bottles by direct matrix-assisted laser desorption ionization time-of-flight mass spectrometry. PLoS ONE.

[15] Lin J.-F., Ge M.-C., Liu T.-P., Chang S.-C., Lu J.-J. (2018). A simple method for rapid microbial identification from positive monomicrobial blood culture bottles through matrix-assisted laser desorption ionization time-of-flight mass spectrometry. J. Microbiol. Immunol. Infect..

[16] Li Y., Gu B., Liu G., Xia W., Fan K., Mei Y., Huang P., Pan S. (2014). MALDI-TOF MS versus VITEK 2 ANC card for identification of anaerobic bacteria. J. Thorac. Dis..

[17] Pandey A., Jain R., Sharma A., Dhakar K., Kaira G.S., Rahi P., Dhyani A., Pandey N., Adhikari P., Shouche Y.S. (2019). 16SrRNA gene sequencing and MALDI-Tof Mass Spectrometry based comparative assessment and bioprospection of psychrotolerant bacteria isolated from high altitudes under Mountain Ecosystem. SN Appl. Sci..

[18] Ratcliffe P., Fang H., Thidholm E., Boräng S., Westling K., Özenci V. (2013). Comparison of MALDI-TOF MS and VITEK 2 system for laboratory diagnosis of Granulicatella and Abiotrophia species causing invasive infections. Diagn. Microbiol. Infect. Dis..

[19] Marín M., Garcia-Lechuz J.M., Alonso P., Villanueva M., Alcalá L., Gimeno M., Cercenado E., Sánchez-Somolinos M., Radice C., Bouza E. (2012). Role of universal 16S rRNA gene PCR and sequencing in diagnosis of prosthetic joint infection. J. Clin. Microbiol..

[20] Ince A., Tiemer B., Gille J., Boos C., Russlies M. (2002). Total knee arthroplasty infection due to Abiotrophia defectiva. J. Med. Microbiol..

[21] Cassir N., Grillo J.C., Argenson J.N., Drancourt M., Levy P.Y. (2011). Abiotrophia defectiva knee prosthesis infection: A case report. J. Med. Case Rep..

[22] Wan J., Larsen M.P., Panwalkar P., Mofidi A. (2020). Simultaneous bilateral revision total knee arthroplasty following Abiotrophia defectiva infection. BMJ Case Rep..

[23] Kocazeybek E., Demirel M., Ersin M., Ergin O.N., Sadic B., Yavuz S.S., Asik M. (2020). Abiotrophia defectiva as a Rare Causative Agent of Periprosthetic Total Knee Arthroplasty Infections: A Case Report and Literature Review. J. Lab. Physicians.

[24] Young J.N., York J. (2022). Abiotrophia Causing Prosthetic Joint Septic Arthritis. Cureus.

[25] Szymczak Z., Michalski P., Dudek J., Płusa T., Baranowski P., Burczy M., Burczy J. (2019). Finegoldia magna the cause of hip revision surgery—A two case report. Pol. Merkur. Lekarski..

[26] Walser F., Prinz J., Rahm S., Zingg P.O., Mancini S., Imkamp F., Zbinden R., Achermann Y. (2022). Antimicrobial susceptibility testing is crucial when treating Finegoldia magna infections. Eur. J. Clin. Microbiol. Infect. Dis..

[27] Clesham K., Hughes A.J., O’ hEireamhoin S., Fleming C., Murphy C.G. (2018). Second-site prosthetic joint infection in patients with multiple prosthetic joints. Eur. J. Orthop. Surg. Traumatol..

[28] Mercurio M., Sanzo V., Rava A., Galasso O., Gasparini G. (2021). Spondylodiscitis After Endovascular Aortic Repair Due to Noninvasive Listeriosis: A Case Report. JBJS Case Connect..

